# CLINE: a web-tool for the comparison of biological dendrogram structures

**DOI:** 10.1186/s12859-019-3149-y

**Published:** 2019-10-28

**Authors:** Rodolfo S. Allendes Osorio, Lokesh P. Tripathi, Kenji Mizuguchi

**Affiliations:** Bioinformatics Project, NIBIOHN, 7-6-8 Saito-Asagi, Ibaraki-shi, Osaka, 567-0085 Japan

**Keywords:** Visualization, Dendrograms, Clustering

## Abstract

**Background:**

When visually comparing the results of hierarchical clustering, the differences in the arrangements of components are of special interest. However, in a biological setting, identifying such differences becomes less straightforward, as the changes in the dendrogram structure caused by permuting biological replicates, do not necessarily imply a different biological interpretation. Here, we introduce a visualization tool to help identify biologically similar topologies across different clustering results, even in the presence of replicates.

**Results:**

Here we introduce CLINE, an open-access web application that allows users to visualize and compare multiple dendrogram structures, by visually displaying the links between areas of similarity across multiple structures. Through the use of a single page and a simple user interface, the user is able to load and remove structures form the visualization, change some aspects of their display and set the parameters used to match cluster topology across consecutive pairs of dendrograms.

**Conclusions:**

We have implemented a web-tool that allows the users to visualize different dendrogram structures, showing not only the structures themselves, but also linking areas of similarity across multiple structures. The software is freely available at http://mizuguchilab.org/tools/cline/. Also, the source code, documentation and installation instructions are available on GitHub at https://github.com/RodolfoAllendes/cline/.

## Background

Clustering, as a technique to organize and reveal the inner topology of data is widely used in biological research and bioinformatics. The reviews of Andreopoulos and colleagues[1] and more recently Xu and Wunsch [2] show examples of clustering being applied to studies that range from gene expression data, to medical imaging.

A detailed description of clustering algorithms is outside the scope of this study, however if required, the reader is encouraged to look at the review made by Xu and Tian [3].

Yet despite its popularity, the use of clustering routines is not free from difficulties, as Ronan and colleagues have reported [4]. In their article, the authors have identified three main issues when dealing with clustering biological data: (1) the high-dimensional nature of the data itself; (2) the need for considering the results of different clustering algorithms; and (3) the difficulties in meaningful interpretation of the clustering output. We believe that, especially when considering points (2) and (3), visualization is a powerful tool in overcoming these problems.

The use of *Dendrograms* is common when considering the visual representation of hierarchical data, and clustering results in particular. A dendrogram is a specific kind of a tree, in which a step forward in the topology represents the division of a node into (typically two) sub-categories. Visually, dendrograms are generally displayed on a two-dimensional plane, with their root on one side of the diagram, and their branches extending in a single direction all the way to the leaves^1^.

Tools for the visualization of dendrograms are widely available, both in terms of stand alone software tools such as Dendroscope [5], or as part of analysis suite such as R [6].

Additionally, specialized visualization tools have also been developed for the specific problem of dendrogram (and thus clustering) comparison. Within the R environment, Dendextend extends the basic display of dendrograms to include pair-wise comparison of structures [7]; while outside R, XCluSim is a tool specifically developed to make it easier for researchers to examine multiple clustering results from a single dataset [8].

In the context of biological research, the study of phylogenetic trees is the area that perhaps has had most influence on the development of tools for the visualization of dendrogram structures.

An early example of this trend is TreeJuxtaposter [9], where authors faced with the problem of comparing large phylogenetic trees, chose to use color to display the structural differences between them. More recently, the web application Phylo.io [10] has been released with the objective of providing an easy to use interface for the visualization and comparison of large phylogenetic trees. Phylo.io allows the comparison of two distinct structures, using color to highlight the differences in the topology, whilst at the same time allowing the user to highlight a node in one tree, to find the best corresponding node on the second tree.

The development of these tools is complemented with efforts in developing more generalized ideas around the problem of phylogenetic tree comparison. For example, Zainon and colleagues [11] take the approach of defining a framework for the display of tree differences, based on the ideas of automatic tree and node positioning, in order to provide a best alignment of similarities, whilst using color for the highlighting of the differences. On the other hand, the Phytools package in R [12], the Environment for Tree Exploration (ETE) Application Programming Interface (API) in Python [13] and the phylotree.js library in JavaScript [14] are available as starting backbone for individuals who wish to develop their own custom solutions or perform specific analysis.

And yet despite the developments in the area, we believe specific issues remain when dealing with the visualization and comparison of dendrograms.

Let us consider the case of toxicogenomics, that is a part of the overall drug discovery process, that focuses on studying the safety of compounds using gene expression profiles.

Usually, data used in toxicogenomics analysis includes multiple measurements of biological processes, such as gene/protein activities, to characterize potentially toxic substances. We call these multiple readings *replicates*.

When the results of different clustering algorithms produce clusters that join together replicates, we can consider these clusters to be equal, regardless of the differences they might have in their branching. Clusters can even be considered equal when they differ in size, as long as they retain the same types of replicates.

It is clear to us that researchers working under these types of conditions, would benefit from a tool that allows the user to quickly identify clusters that, even when not isomorphic, can be interpreted as having the same biological meaning. All previously mentioned tools do provide effective ways to compare structures, both in terms of composition and topology, however, to the best of our knowledge, all these comparisons are based in the idea that leaves are uniquely identifiable.

With these aspects in mind, we introduce **CLINE** (Comparison of bioLogIcal deNdrogram structurEs), a single-page, web-tool specifically designed for the visual comparison of dendrograms, with an emphasis on the display of matching “similar” clusters, i.e. topologies across different dendrograms that, even when not isomorphic, can be perceived as having the same biological meaning.

## Implementation

**Fig. 1:**
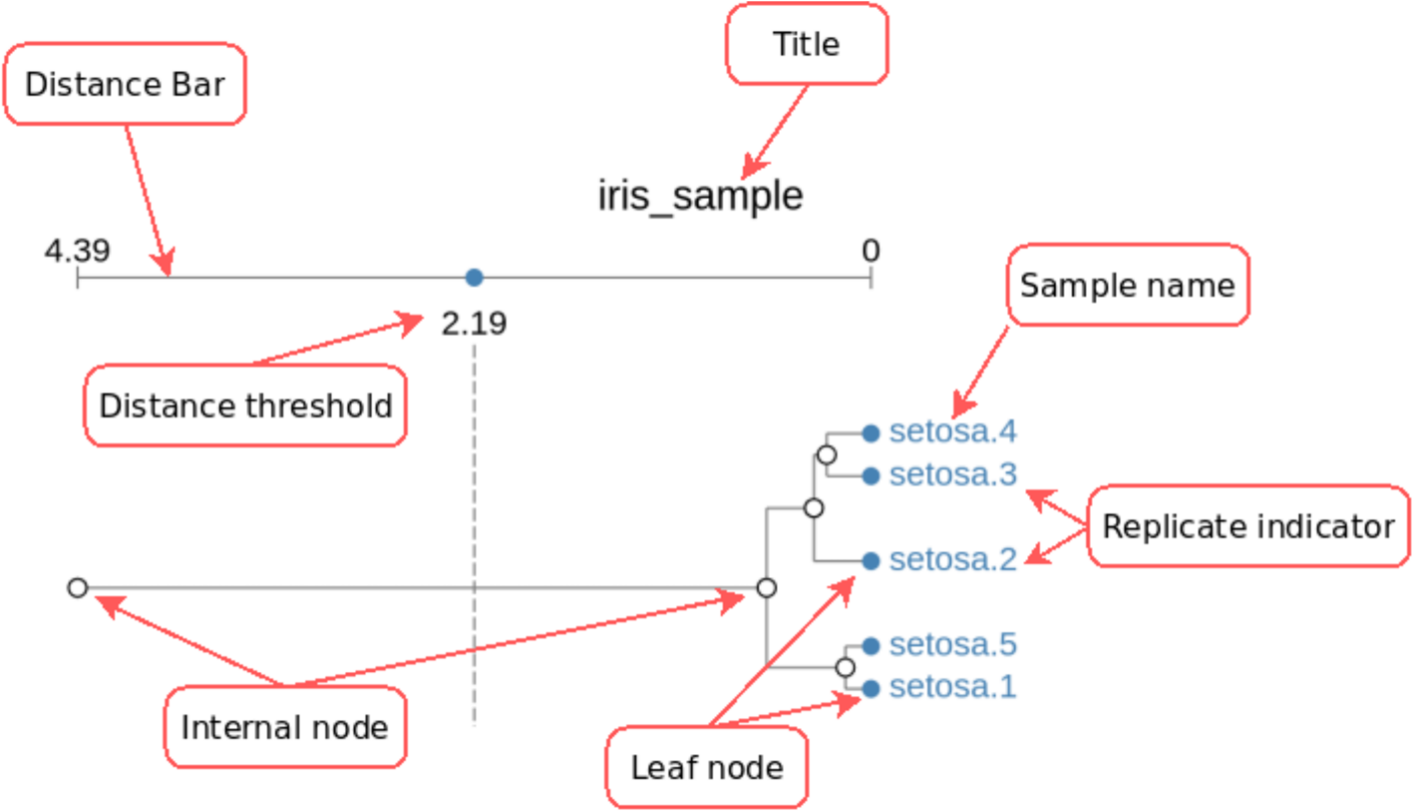
Annotated Dendrogram. Single dendrogram visualization. Clearly distinguishable are the title of the structure, a distance axis and the different nodes and branches between them. Replicate indicators are used to identify multiple instances of a single type of sample (in this case, iris setosa flowers, from the iris dataset)

The main feature of our application is to provide the user with visual cues that identify “similar” sub-trees across different dendrograms. This comparison is performed pair-wise, i.e. pairs of consecutive dendrogram structures within the display are compared to each other, and the results of those comparisons are visually shown. Figure 1 shows a sample dendrogram structure, as displayed by CLINE, annotated with all the significant elements used for finding matches across different structures.

Notice that added to the traditional display, we have included a distance threshold widget. We will use this threshold to constrain the distance (as measured from the leaves) up to which we will search for matches.

Also shown in Fig. 1 are the *replicate indicators* which allow the user to identify multiple replicates of a single type of sample (in the example, five samples of iris setosa) or, in the context of toxicogenomical data, multiple measurements of a single compound from different individuals.

### Dendrogram comparison

In order to display similar clusters across different structures, we first need to define what we understand as ‘similar clusters’. We base this definition on the potential differences that can arise when comparing two clusters.

We consider that clusters might differ in two ways:

1. In terms of their members. Either the type or number (or both) of leaves in each cluster may vary.
2. In terms of their structure. Even if clusters have exactly the same members, the way in which the nodes are connected to each other to form a rooted structure may vary.

By combining the way in which these differences can be found, we have defined three different levels of similarities for the clusters that, even when not equal, can be found to have an equivalent biological meaning. We name these three levels of similarities *Bio-Isomorphism, Re-arrangement* and *Containment*.

#### Bio-isomorphic clusters

We define two clusters as being bio-isomorphic when they have the same topology, i.e. the number of nodes and the branches than join them together are the same on both the clusters. Also, the number of replicates of each type is equal in both the clusters.

**Fig. 2:**
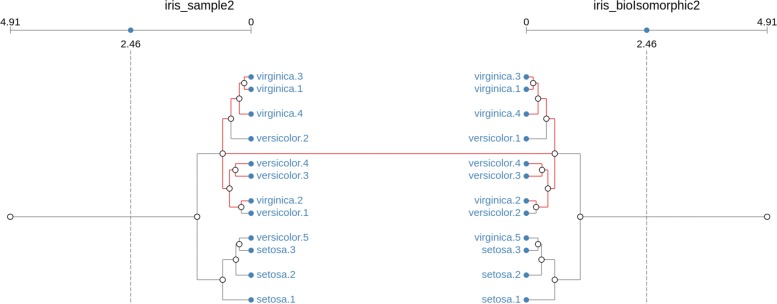
Bio-isomorphic match. Clusters are equivalent if they retain their structure (branches) and the type and number of members (leaves) even if the replicates are found in different places. In this example, bio-isomorphism is retained after versicolor replicates 1 and 2 have switched their positions

However, the position at which the replicates are located within each cluster can change. Figure 2 shows an example of two bio-isomorphic clusters, generated from a selected set of samples of the iris dataset^2^.

From the image, it is clear that the only difference between the bio-isomorphic clusters is the position of replicates versicolor.1 and versicolor.2.

#### Re-arranged clusters

**Fig. 3:**
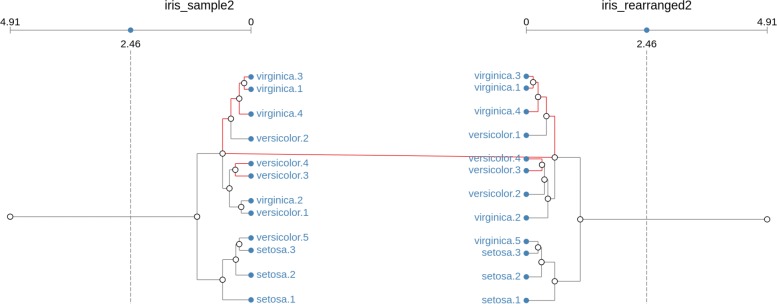
Re-arranged match. Clusters are equivalent if the type and number of members (leaves) is retained, regardless of changes in their structure (branches). In the example, cluster are a match even when the organization of the subcluster containing versicolor replicates 1 (replaced for replicate 2 in the right-hand side structure), 3 and 4, together with virginica replicate 2 changes

Starting from the bio-isomorphism definition, we define re-arranged clusters as those that still retain the same number and type of replicates, but allow them to be connected in different ways. That is, apart from having replicates in different positions, the branches in the clusters are also allowed to be different. Figure 3 shows an example of two re-arranged clusters, generated from samples of the iris dataset.

It is possible to notice that, apart from the permutation of replicates versicolor.1 and versicolor.2, also the structure that joins them together with replicates versicolor.3, versicolor.4 and virginica.2 are different.

#### Contained clusters

**Fig. 4:**
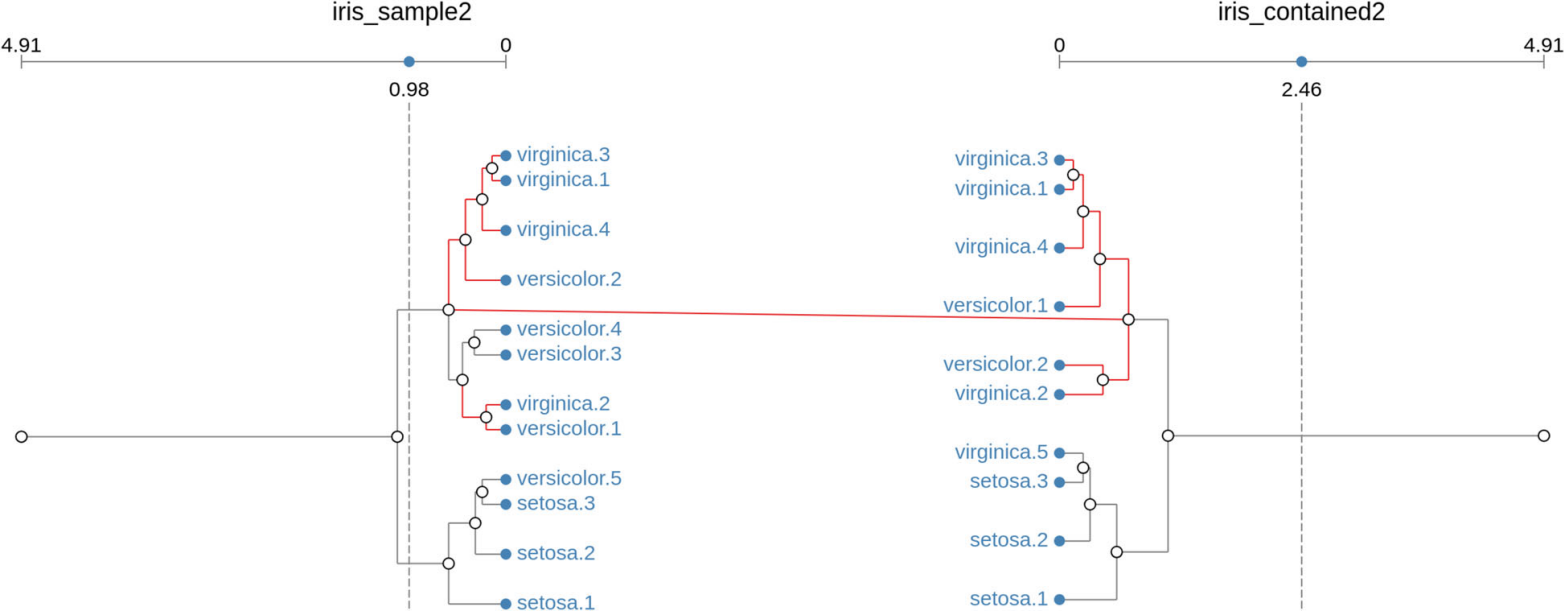
Contained match. Clusters are equivalent as long as they have the same type of replicates, even if their number and by extension their topology, is different. In this example, clusters are matched even when versicolor replicates 3 and 4 are missing in the right-side dendrogram

Finally, we define a cluster to be contained by another when it contains the same type of replicates, but their numbers are allowed to be different. Notice that, since the number of nodes on matching clusters is not retained, the topology (branches) is also different. Figure 4 shows an example of two contained clusters, generated from samples of the iris dataset.

In this final example, replicates versicolor.3 and versicolor.4 have been removed from the right-hand side dendrogram, but even in this case, the clusters are matched, as regardless of the structure, they are both composed of the replicates of types versicolor and virginica.

### Cluster matching implementation

Having previously defined the different types of cluster matchings that our application is set to find and display, now we describe the algorithms that we use for such purpose.

The core of the application’s matching of clusters relies on performing two steps for each pair of consecutive dendrograms: (1) Label the internal nodes of each dendrogram; and (2) Find equal labels across the structures.

The labeling of nodes is based on the idea that leaves already have an assigned label, usually understood as an identifier and replicate information (as shown in Fig. 1). By traversing the dendrogram structure using a post-order strategy, i.e. a bottom-up traversal where all children are processed before processing the parent node, it is possible to construct a label for each internal node in the dendrogram. The algorithm used to label the internal nodes is presented in Algorithm 1.


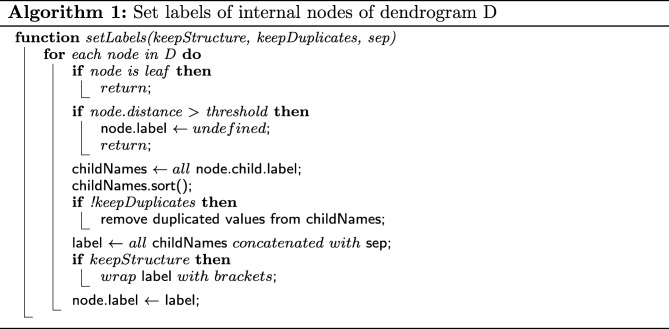


Notice that the traversal of the dendrogram needs to be done following a depth-first approach, to make available the labels of all the children in the current node, before it can be processed. Also, although looped instructions are needed for the processing of each node, for example, when retrieving all child labels, sorting them and filtering the duplicates; clustering results tend to produce binary structures (nodes have only two children), thus the labeling algorithm can be considered to have an *O*(*n*) complexity, where *n* is the number of nodes in the structure.

*keepDuplicates* and *keepStructure* are parameters used to customize the constructed labels. By modifying them, we can implement the three different types of cluster matching described in the previous section, without changing the matching algorithm. For example, by removing duplicated replicate types, we ensure that cluster are matched regardless of the number of replicates for a given type on each dendrogram, thus ensuring a *contained* match.

The second element central to CLINE’s cluster matching strategy is the actual matching of labels across different dendrograms. Algorithms 2 and 3 describe the way in which the matching of labels is performed.


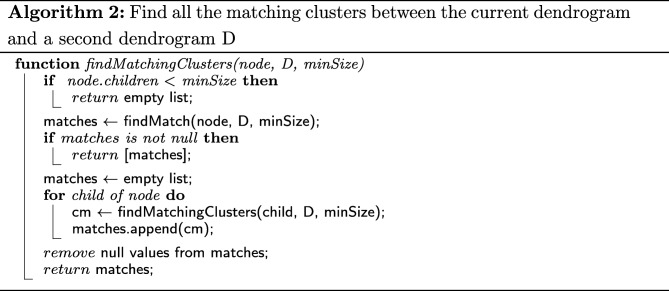


To find all the matching clusters between two dendrograms, the method findMatchingClusters is called using the root of the first dendrogram as *node*, and the second dendrogram as *D*.

Notice that, when a match is found at the current level, it is returned. However, if no match is found, then matches for each of the current node’s children are recursively searched for. By following this breadth-first approach, we ensure that matches are found at the highest level in the source dendrogram.

As the matching results are obtained from the recursive calls of the procedure, these are combined into a single list of matches to be returned at the end of the execution.


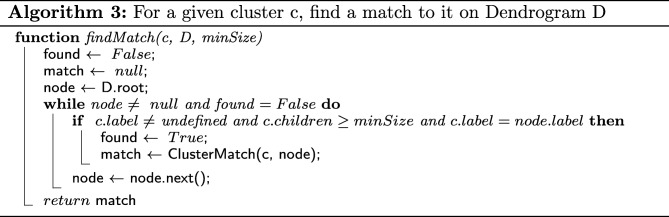


Whilst FindMatchingClusters allows us to recursively traverse the nodes in the source dendrogram, the calls to procedure FindMatch allow us to traverse the target dendrogram. Together, they ensure that for each cluster rooted at a given node in the source dendrogram, we will search a matching cluster, rooted at any node in the target dendrogram.

In order to find the highest match to cluster *c* in dendrogram *D*, we also need to ensure that the traversal through the target dendrogram is performed using a breadth-first approach. We achieve this by appropriately implementing the call to *node*.*next*().

As for the complexity of the overall cluster match search strategy, since we are potentially traversing the target dendrogram for each node in the source dendrogram, we expect the whole process to be *O*(*n*×*m*) in the worst case, where *n* is the number of nodes in the source dendrogram and *m* is the number of nodes in the target dendrogram.

From the definition of the algorithms for node labeling and cluster matching, it can be seen that two additional parameters are used to restrict the search, namely *Distance Threshold* (included in the labeling of nodes) and *Sample Size* (at the time of cluster matching):

- Distance Threshold: The distance threshold defines the distance, measured from the leaves, up to which the internal nodes are considered for matching. All nodes placed above this threshold will be excluded from the cluster matching process.
  By default, the distance threshold for a loaded dendrogram is set to be the half-way point between the leaves and the root of the structure.
  The distance threshold is set individually for each loaded dendrogram, through the corresponding widget, shown in Fig. 1.
- Sample Size: This value defines the number of leaves a cluster needs to have before it is considered as suitable for matching.
  The default sample size used in the application is 3, although typically, we would expect the user to set this value to match the number of replicates in their dataset.
  Sample size is treated as a global parameter, and thus is set at the same time for all dendrograms.

### CLINE’s workflow

CLINE is designed to be user driven, that is, it responds to different types of user interaction in order to display a series of dendrogram structures and the matches found between them.

**Fig. 5:**
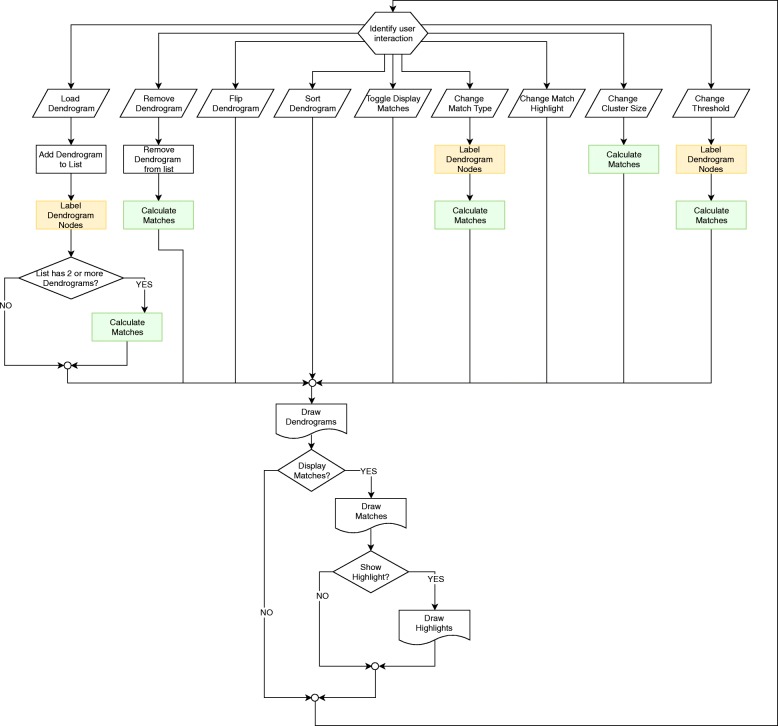
CLINE’s flow-diagram. Being a user interaction guided tool, CLINE constantly wait for the user to interact with it. Once the user performs an action, CLINE follows a series of steps to (re)draw the structures and their matches. Highlighted are the core aspects of the application, the labeling of internal nodes, and the matching of clusters across dendrogram structures

The overall workflow of CLINE is shown in Fig. 5. Notice that, as soon as the user interacts with the application, an entire re-draw cycle is performed. Highlighted in color are the two core routines that CLINE defines to search and display clusters similarity across dendrograms, namely, the labeling of internal nodes (yellow) and the matching of clusters (green), both described in the previous section. Also notice that these core routines are only triggered after certain types of user interactions, reducing the computational load of the drawing cycle, and ensuring a better scalabitily of the application.

## Results and discussion

CLINE is implemented as a single-page website, coded on Typescript^3^, using the Angular framework^4^, and thus requires no installation. To handle the visualization of dendrogram structures we use the D3 Javascript library [15], as it provides an easy way to dynamically generate Scalable Vector Graphics (SVG) displays.

CLINE can be used directly by accessing the website at http://mizuguchilab.org/cline/. No user account is required to use the software.

A description site for the tool is also available on http://mizuguchilab.org/tools/cline. This site includes the description of the tool, together with sample data, a User Guide, copies of the source code and its documentation.

Source code for the application is distributed under an MIT (Massachusetts Institute of Technology) License, and it is also publicly hosted on GitHub (https://github.com/rodolfoAllendes/cline).

### User interface

**Fig. 6:**
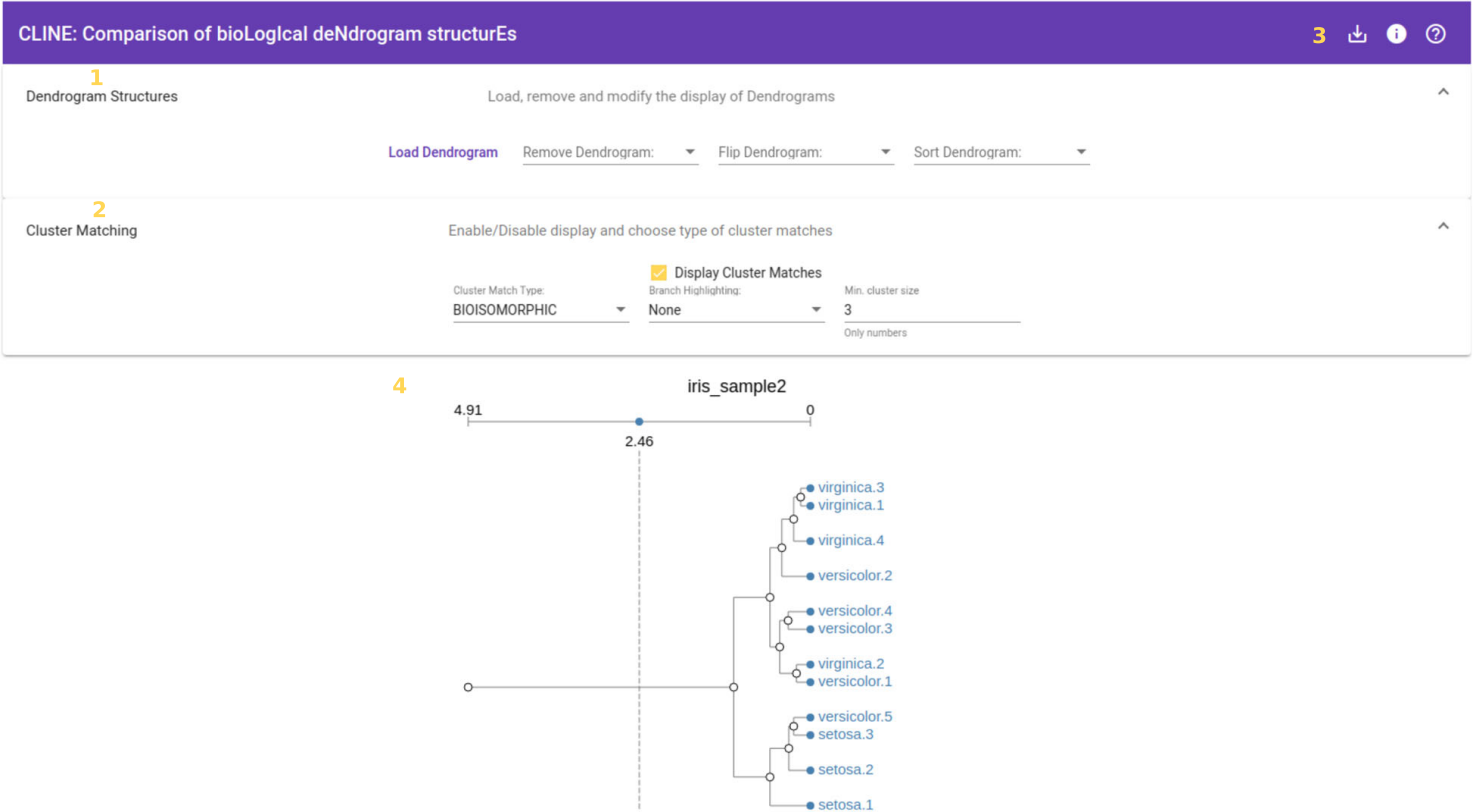
Main interface in CLINE. Four different areas are identified as part of the interface: 1 – Dendrogram manipulation controls; 2 – Cluster Matching controls; 3 – User Menu; and 4 – Visualization panel

Figure 6 shows the interface available to users after accessing CLINE’s website. The interface can be divided into four major components: (1) Dendrogram Controls, (2) Cluster Matching Controls, (3) User Menu, and (4) Visualization Panel. In the following, we give some details on the interaction options available for the first two.

#### Dendrogram controls

Four different types of interactions are defined for the direct manipulation of the dendrograms structures being displayed:

- Load Dendrogram: Allows the user to add a new dendrogram structure to the display area, through the provision of a text file written using the Newick tree format, with distances and leaves names^5^.
  Loaded dendrograms are added to the visualization panel to the right of all the currently displayed structures, expanding as long as necessary to accommodate all structures.
  The vertical size of the display panel, is also scaled to allow a minimum fixed separation space between leaves. The structure with the highest number of leaf nodes determines the height of the visualization panel.
- Remove Dendrogram: Allows the user to remove a single dendrogram structure, by selecting its title from the corresponding drop-down menu.
- Flip Dendrogram: Through flipping, the user is able to alter the horizontal orientation of a structure, that is, to place the leaves where previously the root was located and vice versa.
  An additional *vertical flip* to any (sub-)branch of a dendrogram, simply by clicking the corresponding node.
- Sort Dendrogram: Allows the user to alphabetically sort the leaves of a dendrogram, using a best fit approach, that prevents changing the actual clustering and avoids the introduction of line-crossings in the display.

#### Cluster matching controls

Five types of cluster matching controls are available to the user, allowing him to modify the way in which matches are both calculated and displayed.

- Display Matches: Allows the user to toggle the display of matches.
- Cluster Match Type: Allows the user to select which of the three different types of cluster matching strategies should be applied, namely Bio-Isomorphic, Rearrangement or Containment.
- Branch Highlighting: Using the same color selected to display a given cluster match, it highlights branches within the cluster that are equal or different, depending on user selection. Can be turned off by selecting the value None.
- Minimum Cluster Size: Allows the user to select the minimum number of leaves a cluster must have to be considered for matching. Needs to be greater or equal than 2.
- Distance threshold: Available in the visualization panel for each dendrogram, it allows the user to set the height, from the leaves, up to which cluster matches will be searched.

For more details on the use of each feature, a “User Guide”, available for download from the user menu of CLINE is available.

### Use case: open TG-gates

The Open TG-GATEs (Toxicogenomics Project - Genomics Assisted Toxicity Evaluation System) is a comprehensive collection of gene expression profiles and toxicological data derived from rat experiments on measuring the exposure to different chemical compounds at different dosages and time points [16].

To demonstrate the potential of CLINE in studying data such as the one provided by Open TG-GATEs, we created a dataset comprising of genes that were defined as differentially expressed between the transcriptomes of four (of the 170) compounds that were associated with inflammation (and associated) pathologies- namely lornoxicam (LNX), naproxen (NPX), meloxicam (MLX) and idomethacin (IN). For brevity we focused only on the 9hr, low and high dosage datasets.

Next, we hierarchically clustered the differentially expressed gene profiles by using the pvclust function in R, and “average” and “complete” linkage clustering algorithms together with multiscale bootstrap sampling (10,000 replications).

The resulting dendrogram structures were exported into text files with the Newick tree format using function as.dendrogram, available from the phylogram package in R. The files, named OTG-average.txt and OTG-complete.txt are also available for download through the CLINE website and are included in the *sample-data* folder of the CLINE repository.

**Fig. 7:**
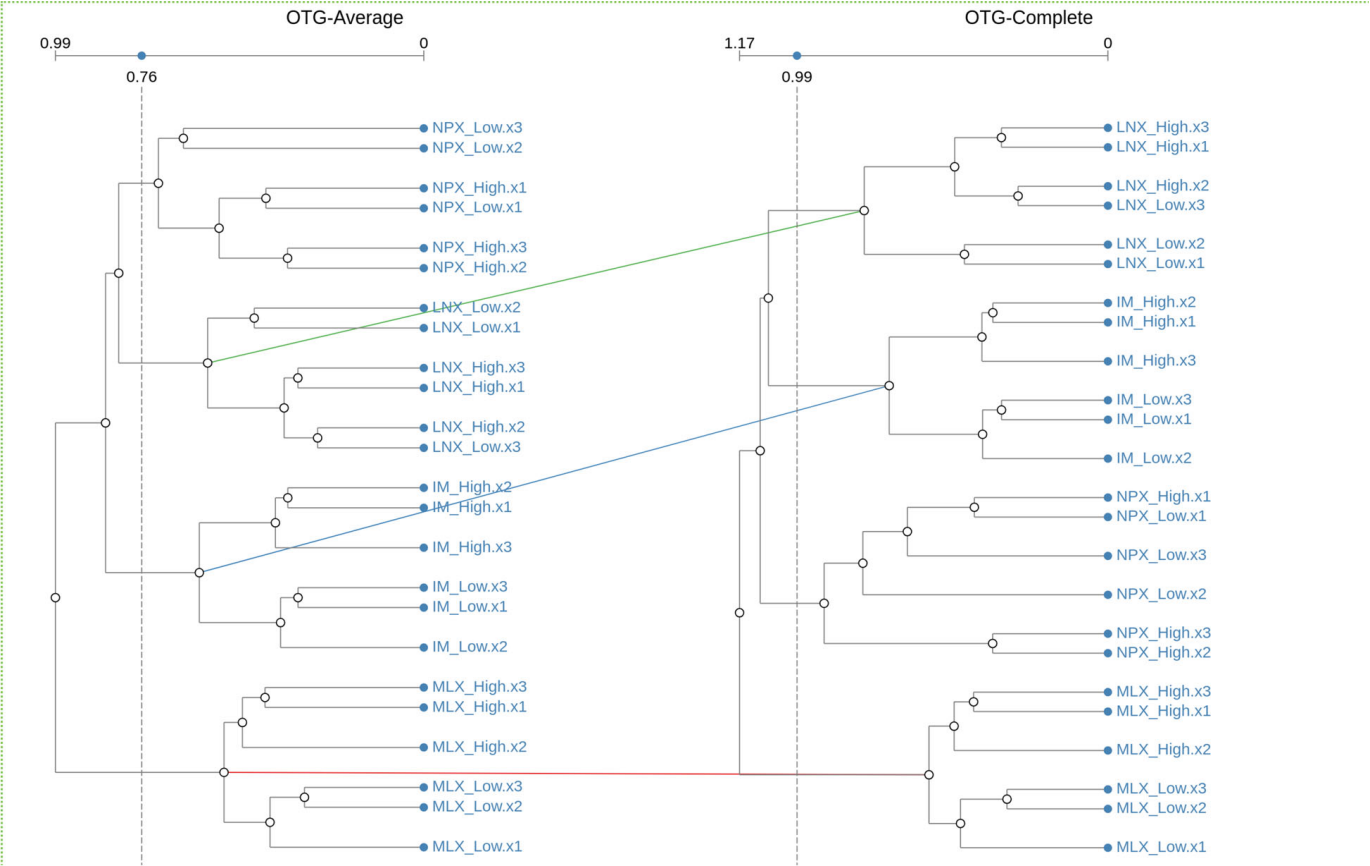
Default OTG Data Visualization Initial visualization of Open TG-GATEs dataset using average and complete linkage clustering algorithms. Clearly visible are the matches between cluster for three of the four components in the dataset. Individual colors are used to identify each match

Figure 7 shows the result of simply loading both files into CLINE. From the figure, it is clear, as it would be expected, that clusters made up from different samples of the same component are matched across the different structures, with the only exception of naproxen (NPX). Notice that the threshold distance has been moved to the left on both dendrograms to allow the match of clusters that contain all samples of each compound.

**Fig. 8:**
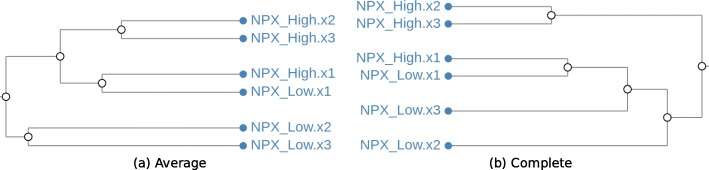
NPX Cluster comparison Comparison of the NPX cluster using Average (**a**) and Complete (**b**) linkage clustering algorithms. Leaves in both clusters are equal, but the branches that link them are different

On closer inspection, it is possible to notice that (as shown in Fig. 8), although all samples of the NPX compound are clustered together, the way in which the two algorithms join them is different. Clearly, we could argue that the clusters are biologically equal, but this is not found by a traditional isomorphic matching strategy, nor by applying a bio-isomorphic strategy for cluster matching.

**Fig. 9:**
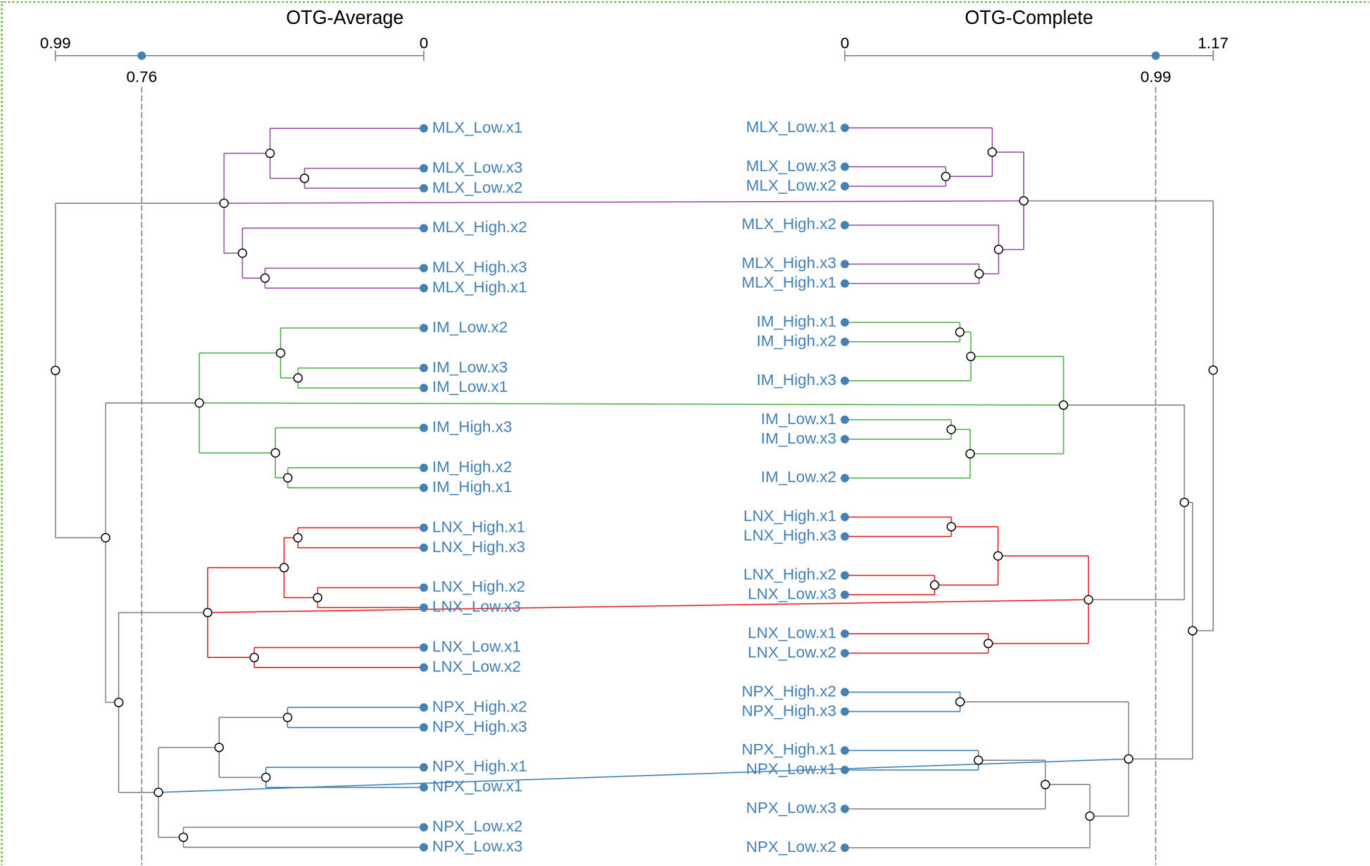
OTG Dataset with Rearranged Cluster Matching Visualization of Open TG-GATEs dataset using Re-arranged matching of clusters. OTG-Complete dendrogram has been flipped and branches sorted to improve spatial positioning of the matching clusters. Equal branches are highlighted. Individual colors are used to identify each match and the corresponding branches

By relaxing the conditions of the cluster matching algorithm, it is possible to automatically find such matching topology. In CLINE, we achieve this simply by changing the type of matching from *Bio-Isomorphic* to *Re-arranged*. Figure 9 shows the result of such selection when applied our Open TG-GATEs dataset.

A highlight to the equal branches on matching clusters is also applied to better identify the point at which the two algorithms differ in their results.

### Pilot user evaluation

In order to get insights into the usability of CLINE, the degree of understanding of the defined strategies for cluster matching, and the overall purpose of CLINE as a dendrogram structure comparison tool, we conducted a pilot, unstructured, qualitative user evaluation.

Two volunteers, with experience in the areas of software development applied to biological contexts, but not necessarily with a background formation in biological sciences took part in this pilot evaluation. Participants had no previous experience in working with CLINE, nor had read any documentation related to the software. No additional demographic information was recorded.

The evaluation was performed remotely, with participants being given a simple document describing a series of tasks to be performed, together with the test files they needed to complete them. No interaction was held during the evaluation.

The tasks performed by the users are described as follows:

- Task 1 - Getting familiar with the Application: In order to get familiar with the interface and the visual representation of dendrograms used by CLINE, users were required to load and remove single structures, and use the different types of flip and sorting controls.
- Task 2 - Exploring comparisons: Users were not given a formal definition of the cluster matching strategies implemented in CLINE, instead, they were given a series of dendrograms pairs for them to load, and explore using, each time, a specific type of cluster match.
- Task 3 - Analyzing Data: For this task in particular, users were requested to replicate the use-case described in Section 1.

After completing Task 2, participants were asked to reply the following questions:

- Is isomorphism (whether two dendrograms are equal to each other) the same as bio-isomorphism? If they are different, what is the difference?
- How is a *rearranged* match, different from a *bio-isomorphic* one?
- How is a *contained* match, different from a *rearranged* one?

And after completing Task 3, participants were asked to reply the following questions:

- How easy/hard is it to use the CLINE?
- How adequate are the visual cues used for the display of matching clusters?
- How easy/hard is it to understand the differences between matching clusters? are the visual cues used to display them adequate?
- Does CLINE meet its purpose?

In terms of usability, the users recognize the tool as easy to use, and value the simplicity and compactness of the user interface. However, they do identify some issues with use of color, particularly when it comes to highlighting branches in a cluster match. As it is not immediately clear what is the criteria used to apply color, this required them extra time to be fully understood.

The highest difficulties where found when it came to the identification cluster matching types. Although this was to be expected, as they had no previous definition of the matching strategies, only by looking at the visual results, they were able to correctly identify the defining properties of bio-isomorphic and contained matches. Re-arranged cluster matching proved to be harder to understand.

Finally, they recognize the usefulness of CLINE for comparative analysis of dendrograms, “as there are many ways to precisely identify equal and different substructures”.

## Conclusions

We have developed a new tool for the visual comparison of dendrogram structures. In our implementation, and through the definition of three different levels of similarity for cluster matches, we believe we provide a suitable platform for biological analysis of clustering methods and results.

Our application matches clusters in the presence of biological replicates, and introduces three different levels of flexibility, required to identify biologically equivalent topology, even when these are different in the traditional comparison sense (i.e. non-isomorphic).

Although only an exploratory pilot evaluation of the software tool was performed, the results we could obtain from it are promising. They also set the base for a thorough user evaluation, to be carried out at a later date.

### Availability and requirements

**Project Name**: CLINE

**Home Page**: http://mizuguchilab.org/tools/cline, https://github.com/rodolfoAllendes/cline

**Operating System(s)**: Windows, MacOS and Linux

**Programming Language**: Typescript

**Other Requirements**: Node and dependencies (only for installation)

**License**: MIT

**Any restrictions to use by non-academics**: None

All sample datasets used for this article are provided in a bundled directory, that can be found both at the tool’s homepage, and at the GitHub repository.

## Data Availability

The datasets generated and/or analysed during the current study are available in the GitHub Repository and the Projects’s Homepage, https://github.com/rodolfoAllendes/clinehttp://mizuguchilab.org/tools/cline.

## Abbreviations

API: Application programming inteface
CLINE: Comparison of bioLogIcal deNdrogram structurEs
IN: Idomethacin
LNX: Lornoxicam
MIT: Massachusetts Institute of Technology
MLX: Meloxicam
NPX: Naproxen
Open TG-GATEs: Toxicogenomics Project Genomics Assisted Toxicity Evaluation system
SVG: Scalable vector graphics

## Footnotes

1 Also common is to make circular displays of dendrograms, where the root is at the centre of a circle, and the branches extend out from the centre.

2 The iris dataset is a well known sample dataset in clustering and machine learning, and can be found at https://archive.ics.uci.edu/ml/datasets/iris

3
https://www.typescriptlang.org/index.html

4
https://angular.io/

5 The Phylogram package in R provides a way to export dendrogram structures text files using the Newick tree format. This is the package used throughout our work to generate our dendrogram files. Other tools suitable for generating dendrograms in this format are: Phylot (https://phylot.biobyte.de/) and the newick builder from Trex-online (http://www.trex.uqam.ca/newick_builder3/index.html)
